# The Impact of Changes in Clinical Guideline on Practice Patterns and Healthcare Utilizations for Kawasaki Disease in Japan

**DOI:** 10.3389/fped.2020.00114

**Published:** 2020-03-24

**Authors:** Yusuke Okubo, Masaru Miura, Tohru Kobayashi, Naho Morisaki, Nobuaki Michihata, Hiroki Matsui, Kiyohide Fushimi, Hideo Yasunaga

**Affiliations:** ^1^Department of Epidemiology, UCLA Fielding School of Public Health, Los Angeles, CA, United States; ^2^Department of Social Medicine, National Center for Child Health and Development, Tokyo, Japan; ^3^Department of Clinical Epidemiology and Health Economics, School of Public Health, The University of Tokyo, Tokyo, Japan; ^4^Department of Cardiology, Tokyo Metropolitan Children's Medical Center, Tokyo, Japan; ^5^Division of Clinical Research Planning, Department of Development Strategy, Center for Clinical Research and Development, National Center for Child Health and Development, Tokyo, Japan; ^6^Department of Health Policy and Informatics, Tokyo Medical and Dental University Graduate School of Medicine, Tokyo, Japan

**Keywords:** coronary artery aneurysm, Kawasaki disease, Diagnosis Procedure Combination inpatient database, healthcare utilization, clinical guideline, practice pattern

## Abstract

**Objective:** Previous studies showed the efficacy of glucocorticoids on prevention of coronary artery lesions (CAL) among Kawasaki disease (KD) patients, and clinical guideline for KD in Japan was changed regarding glucocorticoid use in 2012. However, little is known regarding how the guideline change had impacts on healthcare utilizations and clinical outcomes.

**Methods:** We conducted a retrospective observational study using national inpatient database in Japan among KD patients aged under 18 years during 2010–2015. Recent trends in practice patterns were analyzed, and we divided the hospitals into four groups based on glucocorticoid use: (1) consistently using hospital, (2) started using hospital, (3) stopped using hospital, and (4) never using hospital. Then, we compared healthcare utilizations and risks of coronary artery lesions before and after the guideline change.

**Results:** We identified 24,517 inpatients with KD. From 2010 to 2014, use of glucocorticoid increased from 8.9 to 17.4% of KD inpatients. All types of hospitals showed reduction in coronary artery lesions, but the reduction was the most prominent in hospitals that started using glucocorticoid therapy after clinical guideline change in 2012 (adjusted OR, 0.22; 95%CI, 0.07–0.68). Also, Glucocorticoid consistently using hospitals, started using hospitals, and never using hospitals showed reductions in hospitalization costs, whereas hospitals that stopped using glucocorticoids after clinical guideline change had elevated healthcare costs as opposed to natural trends observed in other groups. Guideline complying hospitals had the greatest reductions in healthcare costs.

**Conclusions:** The early stage glucocorticoid use could be a cost-saving strategy for treatment for KD patients without increasing risks of CAL.

## Introduction

Kawasaki disease (KD) is an acute systemic vasculitis among children (1–5). KD is one of the most common cause of acquired heart disease during childhood in developed countries, and the devastating sequelae are coronary artery lesions (CAL) (1–5). An early suppression of vessel inflammation is thought to be important to prevent development of CAL (1–5).

The standard initial treatments are high-dose intravenous immune-globulin (IVIG) and aspirin (ASA) for children with KD in the acute phase (2, 5–7). Most of the previous studies indicated that 15–20% of KD patients failed to respond to the initial IVIG and ASA treatments, and the non-responders were more likely to develop CAL than IVIG responders, indicating the further requirement for alternative treatment strategies (8–14). In Japan, randomized controlled studies were conducted, which showed the effectiveness of corticosteroids added to IVIG and ASA in acute phase among only high-risk patients using the scoring systems (15, 16). Based on these results, the 2012 Clinical Guideline for Medical Treatment of Acute Stage Kawasaki Disease in Japan describes recommendation for adding corticosteroid to the IVIG and ASA in acute phase to potential high-risk patients or non-responders by the clinical scoring systems (2). Thus, currently many pediatricians in Japan are expected to apply early initial anti-inflammatory therapy (glucocorticoids to the conventional treatment) for KD patients with high clinical scores (4). However, no previous study has investigated the effects of change in the guideline on the trends in clinical practice patterns and healthcare utilizations at a national level.

Therefore, our study investigated the recent changes in clinical practice patterns and healthcare utilizations among children hospitalized with KD, using a national inpatient database in Japan. Furthermore, we also ascertained how the change in KD guidelines impacted on health care costs and risks of CAL among KD patients at hospital levels.

## Methods

### Study Population and Participation

We extracted hospital discharge records of children under 18 years of age hospitalized with KD using the Diagnosis Procedure Combination (DPC) database, a national inpatient database, between July 1, 2010 and March 31, 2015. The details of the DPC database have been described elsewhere (17). In short, the data in the DPC was obtained from more than 1,000 hospitals, and it covers ~55% of data among all inpatients who were admitted to acute-care hospitals in Japan. The DPC data has hospital discharge records and administrative claims information for about seven million inpatients per year. The database contains information on the following: the patient's primary diagnosis; pre-existing comorbidities on admission; complications and comorbidities during hospitalization; the patients' demographic information such as age, sex, and body weight; procedures and treatments. We imputed missing values of body weight based on ideal body weight as defined by WHO recommendations (18). We obtained the approval for the present study from the Institutional Review Board at The University of Tokyo. The Board waived the requirement for informed consent because of the anonymous nature of the data.

Hospitalizations with KD were identified using the International Classification of Diseases, Tenth Revision code (ICD-10 code, M30.3) as the main diagnosis at admission. We included KD patients who were aged <18 years and those who were treated with initial IVIG for their first-time admissions in order to maximize the diagnostic accuracy and prevent misclassifications of KD.

### Measurements of Variables

Patient demographic consisted of age, sex, weight in kg, month and year of admission, ambulance use, types of hospitals (academic or non-academic), dose of IVIG, use of glucocorticoids, cyclosporine A, infliximab, ulinastatin, plasmapheresis, and the need for intensive care.

The outcomes of interest were total hospitalization and drug costs, development of CAL, readmission, total length of hospital stay in days, total IVIG dose, and additional treatment. Development of CAL was identified using an ICD-10 code (I25.4: aneurysm of coronary vessels) and use of antithrombotic drugs (warfarin, ticlopidine, and clopidogrel), assuming that the ICD-10 code reflected all diagnoses of CAL and that antithrombotic use was for medium- or large-sized CAL.

### Statistical Analyses

We investigated the yearly trends in treatment patterns, risk of CAL and readmission, total hospitalization costs, and length of hospital stay (LOS) from fiscal year 2010 to 2014. To evaluate the trends, we constructed multivariable mixed effects logistic and linear regression models that had fiscal year of admission as an independent categorical variable. All models included fixed effects for patient characteristics and random effects for hospitals to account for clustering.

To capture the secular trends in glucocorticoid use within 2 days of initiation of IVIG for Kawasaki disease, we calculated rate of glucocorticoid use in the initial phase. The rates were calculated based on the number of patients who received glucocorticoids in the initial phase as the numerator and the number of patients who were hospitalized with KD as the denominator. A Poisson regression model was used with the natural log of the number of hospitalized patients with KD by months as an offset, and the scale parameter was added to the model to allow the variance to be proportional rather than equal to the mean to account for the over-dispersion (19). We applied a log-linear spline regression model because we assumed that the rate of glucocorticoid use in the initial phase started increasing on January 2012 (3 months before the first randomized controlled study for the effects of glucocorticoid on prevention of CAL was published) and leveled off after March 2013 (3 months after the change in KD guideline).

**Figure 1:**
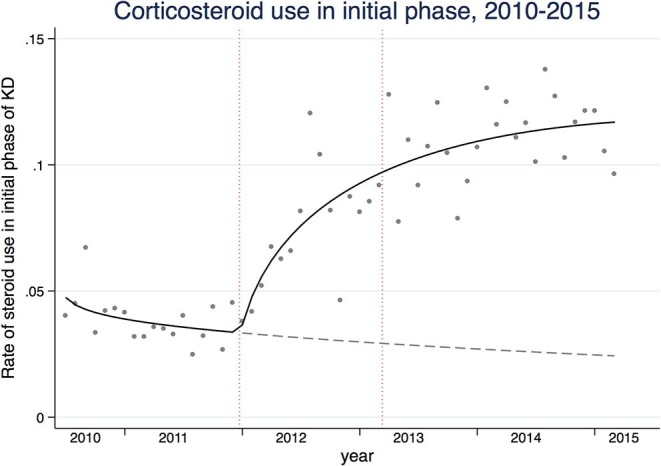
The solid line represents the rates of glucocorticoid use in the acute phase of Kawasaki disease. The gray dashed line represents the counterfactual that would have been observed if the trend were the same before study reports of the efficacy of glucocorticoids and subsequent guideline change. The dashed vertical lines are 3 months before the official publications of the studies (January, 2012) and 3 months after the guideline change (March, 2013).

To investigate the impacts of change in clinical practice guideline on risks of CAL and hospitalization costs at hospital levels, we divided the hospitals into four groups based on glucocorticoid use within 2 days of initiation of IVIG. As glucocorticoid use increased from January 2012 to March 2013 (Figure 1), we considered hospitals that used glucocorticoids during the acute phase of KD before and after the period as consistently using hospitals. Hospitals that never used glucocorticoids during the acute phase before and after the period were considered as never using hospitals. We considered hospitals that never used glucocorticoids during the acute phase before and started using glucocorticoids after the period as started using hospitals. Hospitals that used glucocorticoids during the acute phase before the period and stopped using glucocorticoids after the period were considered as stopped using hospitals. To avoid the potential misclassification of the four types of hospitals, we included patients who were admitted to hospitals that had at least ten patients with KD per year over the 5-year period. After stratifying the hospitals into four categories, we compared total hospitalization and drug costs, risk of CAL, LOS, total mean dose of IVIG, and use of additional treatment (glucocorticoid use more than 3 days after initial IVIG use, cyclosporine A, infliximab, ulinastatin, plasmapheresis) before January 2012 and after March 2013 using mixed effects models. We also used patient characteristics as fixed effects and hospitals as random effects to account for random variations between the hospitals.

The differences in outcomes of interest were estimated with 95% confidence intervals (95%CIs). For the mixed effects linear regression models, log-transformation was required to ensure the error term normality assumption. As the doses of IVIG were missing for 2,345 patients, the analyses relevant to IVIG dose were conducted with the remaining 22,172 patients. We set statistical significance at two-sided *P* < 0.05 for all of the analyses. We used STATA software version 14.1 for all data analyses (StataCorp LP, TX, USA).

## Results

We observed a total of 24,517 children hospitalized with KD over the study period. Table 1 shows the summary of the baseline patient characteristics, treatment patterns, and healthcare utilizations, stratified by fiscal years of admissions. In the mixed effects model analyses, glucocorticoid use for both initial and all phases of KD showed an upward trend (*P*_*trend*_ < 0.001). The spline model showed an increasing trend in glucocorticoid use during the initial phase that started in January 2012 and leveled off after March 2013 (Figure 1). An upward trend in infliximab use was observed (*P*_*trend*_ < 0.001), whereas little changes were observed in dose of IVIG, use of cyclosporine A, ulinastatin, and plasmapheresis. Diagnoses of CALs defined by both ICD-10 and antithrombotic therapy decreased from 4.5 and 1.0% in 2010 to 2.4 and 0.7% in 2014, respectively. Total hospitalization costs and LOS also showed decreasing trends over the study period (*P*_*trend*_ < 0.001).

**Table 1:** Baseline characteristics of children hospitalized with KD by fiscal year of admission and trend analyses for hospital utilizations and adjunctive treatment.

		**Fiscal year of admission**					
	**Total**	**2010**	**2011**	**2012**	**2013**	**2014**	
Total Admissions, *n*	24,517	3,369	4,699	4,700	4,893	6,856	
Age (years), mean (SD)	2.53 (2.0)	2.45 (3.9)	2.46 (4.0)	2.48 (4.1)	2.55 (4.1)	2.65 (4.2)	
Male, *n* (%)	14,146 (57.7)	1,980 (58.8)	2,724 (58.0)	2,690 (57.2)	2,806 (57.3)	3,946 (57.6)	
Ambulance, *n* (%)	508 (2.1)	63 (1.9)	74(1.6)	86 (1.8)	145 (3.0)	140 (2.0)	
Academic, *n* (%)	4,283 (17.5)	588 (17.5)	738(15.7)	835 (17.8)	997 (20.4)	1,125 (16.4)	
Season							
Spring, *n* (%)	5,747 (23.4)	400 (11.9)	1,237(26.3)	1,234 (26.3)	1,324 (27.1)	1,552 (22.6)	
Summer, *n* (%)	6,255 (25.5)	839 (24.9)	1,196(25.5)	1,222 (26.0)	1,249 (25.5)	1,749 (25.5)	
Fall, *n* (%)	5,473 (22.3)	907 (26.9)	981 (20.9)	999 (21.3)	1,020 (20.8)	1,566 (22.8)	
Winter, *n* (%)	7,042 (28.7)	1,223(36.3)	1,285 (27.3)	1,245 (26.5)	1,300 (26.6)	1,989 (29.0)	
	**Total**	**2010**	**2011**	**2012**	**2013**	**2014**	***P**_***trend***_*
IVIG (g/kg), mean (log-SD)	2.32 (0.01)	2.27 (0.01)	2.23 (0.01)	2.37 (0.01)	2.32 (0.01)	2.32 (0.01)	0.37
Glucocorticoid use							
All phases, *n* (%)	3,410 (13.9)	299 (8.9)	405 (8.6)	647 (13.8)	869 (17.8)	1,190 (17.4)	<0.001
Initial phase, *n* (%)	2,007 (8.2)	141 (4.2)	175 (3.7)	381 (8.1)	516 (10.5)	794 (11.6)	<0.001
Other Treatment							
Cyclosporine A, *n* (%)	223 (0.9)	23 (0.7)	29 (0.6)	48 (1.0)	59 (1.2)	64 (0.9)	0.11
Infliximab, *n* (%)	105 (0.5)	5 (0.1)	13 (0.2)	8 (0.2)	27 (0.6)	52 (0.8)	<0.001
Ulinastatin, *n* (%)	955 (3.9)	83 (2.5)	163 (3.5)	202 (4.3)	227 (4.6)	280 (4.1)	0.12
Plasmapheresis, *n* (%)	97 (0.4)	6 (0.2)	11 (0.2)	19 (0.4)	30 (0.6)	31 (0.5)	0.17
Intensive care, *n* (%)	181 (0.7)	18 (0.5)	29 (0.6)	34 (0.7)	50 (1.0)	50 (0.7)	0.59
Outcomes							
Readmission, *n* (%)	397 (1.6)	45 (1.3)	79 (1.7)	70 (1.5)	88 (1.8)	115 (1.7)	0.62
CAL (ICD-10), *n* (%)	725 (3.0)	153 (4.5)	142 (3.0)	137 (2.9)	128 (2.6)	165 (2.4)	<0.001
CAL (drug), *n* (%)	178 (0.7)	34 (1.0)	22 (0.5)	30 (0.6)	44 (0.9)	48 (0.7)	0.043
Cost, JPY	300,619	314,387	310,060	302,964	298,179	288,056	<0.001
LOS, days	10.8	11.0	10.8	10.8	10.8	10.3	<0.001

*SD, standard deviation; CAL (ICD-10), diagnoses of coronary artery lesion based on ICD-10; CAL (drug), diagnoses of CAL based on clopidogrel, ticlopidine, or warfarin use; JPY, Japanese yen; LOS, length of stay*.

We identified 91 hospitals that had at least 10 patients per year during 2010–2014, which included 38 consistently using hospitals, 17 started using hospitals, seven stopped using hospitals, and 29 never using hospitals (Table 2). Table 3 shows the total hospitalization costs and risks of CAL stratified by hospital types based on glucocorticoid use in the initial phase of KD. Total hospitalization costs comparing before January 2012 to after 2013 March were reduced in the always using hospital, stopped using hospital, and never using hospitals. The most prominent reduction was observed in started using hospitals (adjusted difference, −20,295 JPY; 95%CI, −28,865 to −13,448). In contrast, only stopped using hospitals did not show reduction of hospitalization costs (adjusted difference, −10,198 JPY; 95%CI, −15,731 to −4,479). The odds of CAL development were decreased in always using and never using hospitals. The most prominent reduction in odds of CAL was observed in started using hospitals (adjusted OR, 0.22; 95%CI, 0.07–0.68).

**Table 2:** Selection and timing of steroid use at hospital levels.

	**Before January 2012, hospital (%)**		
	**Non-initial use or never use**	**Initial use**	**Total**
	**46**	**45**	**91**
**After March 2013, hospital (%)**			
Non-initial use or never use	29 (31.9)	7 (7.7)	36
Initial use	17 (18.7)	38 (41.8)	55

**Table 3:** Differences in total costs and proportions of CAL between before 12/2011 and after 4/2013 stratified by use of steroid use defined at hospital levels.

**Hospital characteristics**	**Always use**	**Started use**	**Stopped use**	**Never use**
Number of patients, *N*	4,029	1,544	624	2,483
Number of hospitals, *N*	38	17	7	29
**Total hospitalization cost**				
Before 12/2011, mean JPY (log-SD)	301,374 (0.5)	289,294 (0.5)	304,882 (0.6)	286,081 (0.6)
After 4/2013, mean JPY (log-SD)	290,259 (0.5)	266,495 (0.5)	321,142 (0.6)	279,568 (0.5)
Adjusted Difference, JPY (95%CI)	−11,270 (−15,546, −6,887)	−20,295 (−26,865, −13,448)	7,926 (−5,838, 22,856)	−10,198 (−15,731, −4,479)
**CAL**				
Before 12/2011, *N* (%)	59 (3.5)	11 (1.9)	11 (3.0)	33 (3.2)
After 4/2013, *N* (%)	44 (1.9)	5 (0.5)	4 (1.5)	27 (1.9)
Adjusted OR (95%CI)	0.54 (0.35, 0.82)	0.22 (0.07, 0.68)	0.52 (0.15, 1.77)	0.50 (0.29, 0.86)

*JPY, Japanese-Yen; Log-SD, log-transformed standard deviation; CAL, coronary artery lesion; CI, confidence interval; OR, odds ratio*.

Table 4 shows the changes in total drug costs, LOS, IVIG dose, and additional treatment use. Reduction in total drug costs were observed in the always using, started using, and never using hospitals, whereas an elevated drug cost was found in stopped using hospitals. All hospitals showed decreases in LOS, and the difference was substantial on stopped using hospitals. Only stopped using hospitals showed an increase in average total IVIG dose (adjusted difference, 0.18 mg/kg; 95%CI, 0.01–0.36).

**Table 4:** Differences in drug costs, mean intravenous immunoglobulin (IVIG) dose, length of hospital stay (LOS), proportions of additional treatment between before 12/2011 and after 4/2013 stratified by use of steroid use defined at hospital levels.

**Hospital characteristics**	**Always use**	**Started use**	**Stopped use**	**Never use**
**Total drug cost, JPY**				
Before 12/2011, mean (log-SD)	284,637 (0.5)	278,098 (0.4)	274,191 (0.6)	275,328 (0.5)
After 4/2013, mean (log-SD)	273,282 (0.5)	265,580 (0.5)	303,979 (0.5)	269,850 (0.5)
Adjusted Difference, (95%CI)	−9,115 (−13,271, −5,238)	−16,092 (−22,140, −9,803)	12,172 (109, 25,151)	−8,688 (−13,737, −3,474)
**Total LOS in days**				
Before 12/2011, mean (log-SD)	10.7 (0.5)	11.8 (0.4)	11.5 (0.4)	10.7 (0.5)
After 4/2013, mean (log-SD)	10.2 (0.5)	11.6 (0.4)	9.3 (0.4)	10.3 (0.4)
Adjusted Difference, (95%CI)	−0.35 (−0.60, −0.09)	−0.30 (−0.72, 0.13)	−2.37 (−2.92, −1.79)	−0.71 (−0.97, −0.46)
**Total IVIG dose per kg**				
Before 12/2011, mean (log-SD)	2.37 (0.01)	2.46 (0.02)	2.35 (0.03)	2.33 (0.02)
After 4/2013, mean (log-SD)	2.37 (0.01)	2.36 (0.02)	2.55 (0.02)	2.34 (0.01)
Adjusted Difference, mean (95%CI)	0.02 (−0.04, 0.09)	−0.03 (−0.09, 0.02)	0.18 (0.01, 0.36)	−0.01 (−0.09, 0.073)
**Additional treatment**				
Before 12/2011, *N* (%)	60 (3.6)	23 (4.0)	25 (6.8)	64 (6.1)
After 4/2013, *N* (%)	101 (4.3)	41 (4.2)	14 (5.4)	118 (8.2)
Adjusted OR (95%CI)	1.31 (0.93, 1.86)	1.06 (0.62, 1.81)	0.79 (0.39, 1.62)	1.23 (0.83, 1.84)

*Additional treatment includes use of glucocorticoid use more than 3 days after initial IVIG, cyclosporine A, infliximab, methotrexate, ulinastatin, and plasma exchange; JPY, Japanese-Yen; Log-SD, log-transformed standard deviation; CAL, coronary artery lesion; CI, confidence interval; OR, odds ratio*.

## Discussion

In the present study, we observed increasing trends in initial phase glucocorticoid use for KD patients, after publications that proved the efficacy of glucocorticoid use for preventing CAL and subsequent KD treatment guideline change. We observed the reduction in hospitalization costs among hospitals that consistently used glucocorticoids, started using after the change in guideline, and never used glucocorticoids. On the other hand, hospitals that stopped using glucocorticoids did not show decreasing trends in hospitalization costs. To our best knowledge, this is the first study that assessed the clinical practice patterns for KD and the impacts of KD guideline change on risks of CAL and healthcare costs at hospital levels.

Recommendations, regarding to adjunctive therapy for primary treatment with IVIG and ASA and additional therapy in the IVIG-resistant case, are slightly different between the clinical guideline in Japan and that in the US (2, 5). First, the risk stratification scores have been proven useful to identify Japanese children in Japan with KD (10, 11), whereas the scores showed low sensitivity and poor negative predictive value outside of Japan (20). Second, recommendations of pulsed methylprednisolone for adjunctive therapy for primary treatment are different between the guidelines in Japan and the US (2, 5). Third, the clinical guideline in Japan describes ulinastatin and plasmapheresis as one of treatment options for additional treatment in the IVIV-resistant cases (2).

An increasing trend in glucocorticoid use was observed over the study period. The trend could be explained by the impacts of previous randomized controlled studies that identified efficacy of glucocorticoids in initial treatment of KD and subsequent change in Japanese KD guideline during 2012 (4, 15, 16). In fact, our study showed that the upward trend started 3 months before publishing the previous studies and leveled off after change in Japanese KD guideline. Correspondingly, the risks of CALs showed a decreasing trend. However, we believe that the decreasing trend in CALs could be multifactorial such as upward trends in glucocorticoid use and other aggressive anti-inflammatory treatment, and earlier administrations of IVIG. Indeed, a nationwide survey of KD in Japan reported that the proportions of IVIG administrations within 5 days increased from 67.7% in 2010 to 69.2% in 2014 (4). Furthermore, slightly upward trends in use of infliximab and ulinastatin were observed in our study, probably because of the guideline recommendations for early aggressive anti-inflammatory therapy and the study results that reported the effectiveness of infliximab and ulinastatin (4, 21–24). We believe that these factors may have contributed to the change in treatment strategy and reduction in risks of CAL, rather than a single effect of glucocorticoid use in the initial phase.

The effects of changes in treatment strategies at hospital levels on the risk of CAL and healthcare costs are uncertain, and finding a cost-saving strategy with improving prognosis is extremely important considering the increasing healthcare expenditure in high-income countries (25). We observed decreasing trends in risks of CAL and healthcare costs among hospitals that used glucocorticoids consistently and those that never used them. As we cited above, we believe that the decreasing trends in the risks of CAL reflected several factors such as increasing use of glucocorticoid, earlier administrations, and elevated doses of IVIG. Furthermore, decreasing trends in healthcare costs in consistently and never using hospitals could be explained by natural trends in healthcare costs among pediatric inpatients in Japan, rather than effects of change in the clinical guideline for KD. In fact, downward trends in total hospitalization costs were already observed for other pediatric disorders in Japan, such as immune thrombocytopenia and respiratory infections (26–28).

Complying with the changes in guideline in terms of glucocorticoid use in the initial phase of KD could be the most effective strategy to save healthcare costs for KD inpatients. Indeed, we observed the greatest reduction in healthcare costs (−20,295 JPY per KD inpatients) among hospitals that started using glucocorticoids compared with consistently using and never using hospitals. The greatest reduction in total hospitalization costs in the started using hospitals could reflect decreases in drug costs and slightly decreased LOS. On the other hand, stopped using hospitals showed slightly elevated healthcare costs, which contradicted to natural trends in reduction of healthcare costs. Considering for the increase in IVIG dose, we believe that the stopping the use of initial phase glucocorticoid use may have increased the cases of non-response to initial IVIG, which resulted in elevated total IVIG dose and subsequent increases in hospitalization costs.

We acknowledge several limitations to our study. The number of KD inpatients and subsequent development of CAL could have been underestimated because of possible underreporting and/or misclassification of ICD-10 code. However, we maximized diagnostic accuracy of KD and CAL by restricting to patients who received IVIG and identified patients with antithrombotic treatment. As a result, our estimates of CAL were mostly similar to the findings from the previously reported national survey in Japan. The detailed clinical presentation, laboratory data, and patient information for the follow-up of KD at outpatient visits were unavailable in the DPC database. Due to these limitations, we were unable to reliably evaluate and compare the difference of pre-treatment severity scores among KD patients, and we could not compare patients between hospitals with different treatment strategies. In addition, we were not able to detect cases of CAL in KD patients that were detected during outpatient follow-up periods. The strength of this study was the use of the DPC database, which is the only national inpatient database currently available in Japan. Using the DPC database, we calculated the robust estimates of the recent clinical practice patterns in KD and the impact of change in KD guideline at hospital levels throughout Japan.

In summary, this study added novel insights into strategies of KD treatment by comparing the different treatment strategies. Glucocorticoid treatment in the initial phase of KD could be a cost-saving strategy with improving clinical outcomes. We believe that our investigations showed valuable information to improve prognosis for CAL and save healthcare expenditure at hospital levels.

## Data Availability Statement

The raw data supporting the conclusions of this article will be made available by the authors, without undue reservation, to any qualified researcher.

## Author Contributions

YO and NMi designed the data collection instruments, coordinated data, drafted the initial manuscript, and performed the initial analyses. KF, HY, MM, TK, HM, and NMo supervised data collection, revised the manuscript, and approved the final manuscript as submitted. Each author listed on the manuscript has seen and approved the submission of this version of the manuscript and takes full responsibility for its contents.

### Conflict of Interest

MM and TK received honorariums from the Japan Blood Products Organization and Teijin Pharma Limited Nihon Pharmaceutical Co. MM received an honorarium from the Mitsubishi Tanabe Pharma Corporation. The remaining authors declare that the research was conducted in the absence of any commercial or financial relationships that could be construed as a potential conflict of interest.
